# A Case of Gastric Metastatic Melanoma 15 Years after the Initial Diagnosis of Cutaneous Melanoma

**DOI:** 10.1155/2018/7684964

**Published:** 2018-07-29

**Authors:** Sohail Farshad, Scott Keeney, Alexandra Halalau, Gehad Ghaith

**Affiliations:** ^1^Internal Medicine Department, William Beaumont Hospital, Royal Oak & Oakland University William Beaumont School of Medicine, USA; ^2^Gastroenterology Department, William Beaumont Hospital, Royal Oak & Oakland University William Beaumont School of Medicine, USA

## Abstract

Melanoma is the most common cancer to metastasize to the gastrointestinal tract; however, metastasis to the stomach is a rare occurrence. We present the case of a patient with a history of melanoma of the chest wall 15 years prior to presentation who initially presented to the hospital with sepsis but was later found to have metastatic melanoma in the gastric cardia. This case illustrates the rare occurrence of metastatic melanoma to the stomach which occurred 15 years after the initial skin diagnosis of melanoma was made, its endoscopic appearance, and how the nonspecific symptoms frequently lead to a delayed diagnosis or one that is not made at all until after autopsy. For these reasons, endoscopy should be promptly performed if there is a suspicion of gastrointestinal metastatic melanoma.

## 1. Introduction

Metastatic melanoma to the stomach is a rare entity and portends a poor prognosis with a median survival of 4 to 6 months [1]. The most common gastrointestinal (GI) metastatic sites from cutaneous melanoma are the jejunum and ileum, followed by the colon, rectum, and then the stomach [2]. The clinical manifestations are usually nonspecific, and many patients are asymptomatic until the disease progresses further, which can delay the diagnosis or miss it entirely until autopsy [3]. Patients may present with symptoms of nausea, vomiting, gastrointestinal bleeding, weight loss, and possibly acute perforation [4]. If there is suspicion for metastasis to the GI tract, esophagogastroduodenoscopy (EGD), colonoscopy, and, if needed, a small bowel investigation with capsule endoscopy should be performed for direct visualization and biopsy should be obtained if a lesion is discovered. Treatment options include surgical resection, immunotherapy, and targeted therapy. Surgical resection can be a palliative intervention if a patient is symptomatic [5], and it can also prolong survival [6].

## 2. Case Report

**Figure 1:**
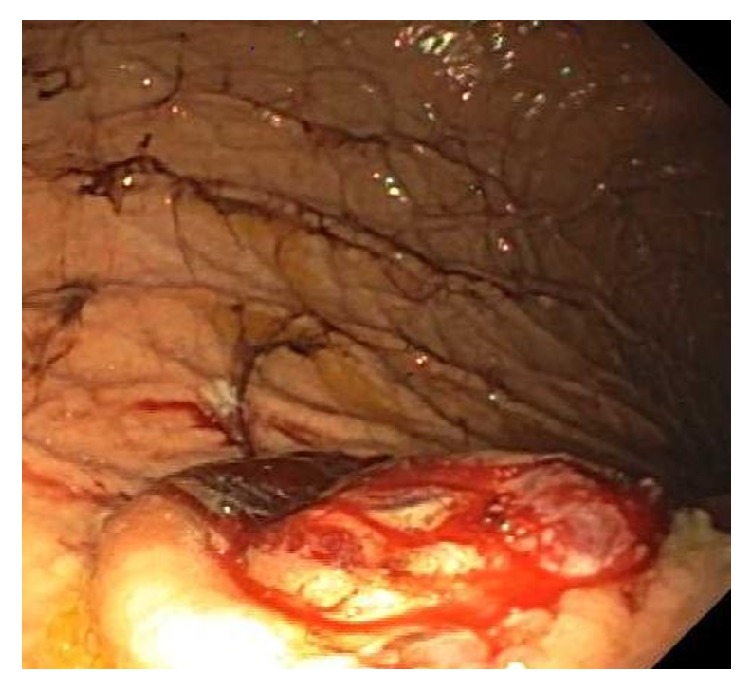
Picture taken from EGD showing a 50mm bleeding mass with an ulcerated center from which biopsies were taken.

We present the case of an 89-year-old male with a history of end stage renal disease on hemodialysis and localized melanoma of the chest status after excision 15 years ago who presented to the hospital complaining of fatigue, rigors, and fever one day after his first ever hemodialysis session. Complete blood count revealed hemoglobin of 7.7 g/dL, white blood cell count of 16.2 bil/L, and platelet count of 195 bil/L. In the emergency department, the patient was febrile measuring 38.2 degrees Celsius, with a blood pressure of 146/85 mmHg, heart rate of 85 beats/minute, and respiratory rate of 19. Chest X-ray showed a 5-centimeter mass in the right upper lobe of the lung. Blood cultures grew Methicillin-resistant Staphylococcus aureus (MRSA), attributed to the recent tunneled central venous catheter as the source of infection. Five days since the date of hospital admission, the patient's hemoglobin acutely decreased to 5.1 g/dL. A fecal occult blood was positive from digital rectal exam. An esophagogastroduodenoscopy showed a 50-millimeter noncircumferential bleeding mass in the gastric cardia, with raised borders and a central, protruding, ulcerated center from which biopsies were taken (Figure 1).

**Figure 2:**
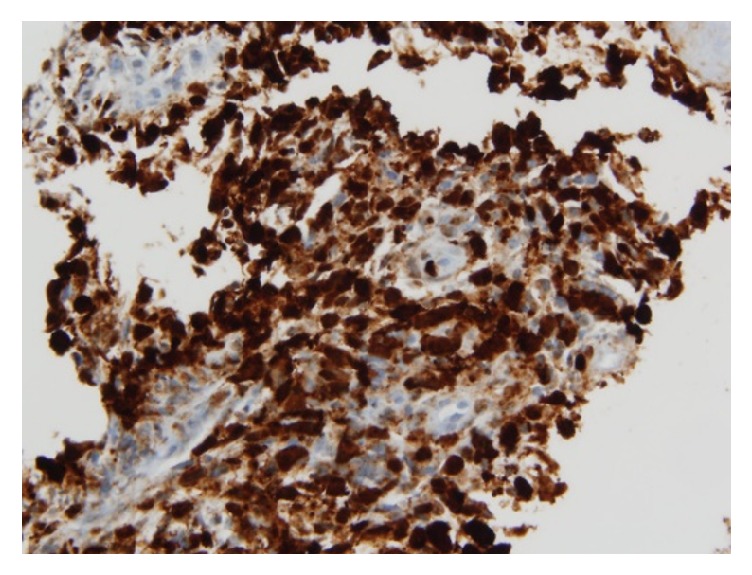
Immunohistochemistry positive for S-100 consistent with metastatic melanoma.

**Figure 3:**
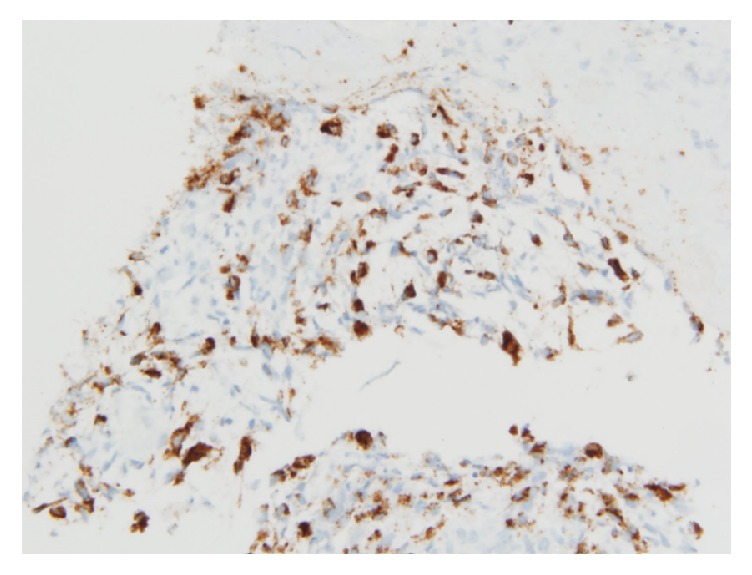
Immunohistochemistry from biopsy positive for HMB-45 consistent with melanoma.

Computed Tomography (CT) scan of the abdomen and pelvis (with oral contrast only) showed a heterogeneous density involving the dome of the liver concerning metastatic disease. The biopsy report revealed a high grade malignant neoplasm with immunohistochemistry positive for cytokeratin CAM 5.2, polytypic cytokeratin, and 4 different melanoma markers (SOX-10, S-100, MART-1, and HMB-45) (Figures 2 and 3). Considering the patient's history of melanoma and biopsy findings, the diagnosis of metastatic malignant melanoma to the stomach was made. Due to his functional status and suspicion for diffuse metastatic disease to the liver and lung, he was not considered a candidate for surgical resection. The patient was started on treatment with nivolumab. Unfortunately, the patient decompensated 2 months after his diagnosis was made and was enrolled in hospice care.

## 3. Discussion

Suspicion for metastatic melanoma to the GI tract should be investigated with an EGD, colonoscopy, capsule endoscopy if EGD and colonoscopy do not find a lesion, and a biopsy if a lesion is found. Initially, CT images should be obtained to search for metastases; however, the sensitivity of CT is only 60-70% [2]. In addition, because melanoma most commonly metastasizes to the small bowel, EGD and colonoscopy may be falsely negative. In these cases, a capsule endoscopy should also be considered as there have been publications that report its utility in finding tumors in the small bowel [7].

The endoscopic appearance of metastatic tumors in the stomach is classified into two groups, which resemble either submucosal or primary gastric tumors (further subdivided into early gastric cancer or advanced gastric cancer) [8]. Those resembling advanced gastric cancers are further subdivided as type 1 (polypoid tumor), type 2 (ulcerated tumor with sharply demarcated margins), type 3 (ulcerated tumor without definite borders), or type 4 (diffusely infiltrating tumor) [8]. Our patient's EGD findings were consistent with the appearance of advanced gastric cancer type 3. However, it is important to note that there are no characteristic features seen on EGD that can identify the etiology of gastric metastases because of the variable morphology of tumors [8]. The diagnosis of metastatic melanoma is made from the biopsy specimen when the immunohistochemistry stain is positive for S-100 and antibody HMB-45 [9].

The literature has shown that gastric metastases usually occur within one year from the time of the primary tumor diagnosis. A previous study illustrated that half of their cases of gastric metastases were found within one year from the time of the diagnosis of the primary tumor (including lung cancer, breast cancer, and melanoma), with the longest period being 48 months [8]. Another study showed 67% of their cases of recurrent melanoma with gastric metastases occurred within 3 years [10]. Our case was rare in that the diagnosis of primary melanoma was made 15 years prior to the time when the diagnosis of gastric metastases was made.

Treatment for metastatic melanoma includes surgical resection, immunotherapy, targeted therapy, and possibly radiation therapy to symptomatic sites. Since immunotherapy and targeted therapy have been developed, cytotoxic chemotherapy is no longer considered as a first-line treatment [11]. If a patient's functional status is acceptable, surgical resection should be considered as it may prolong survival time with fewer side effects compared to chemotherapy; however more studies are needed to evaluate its true benefits, especially regarding gastric tumor resection. One study demonstrated that patients with total excision of intra-abdominal metastases had a 5% postoperative mortality, a median survival of 9.6 months, a 1-year survival of 44%, and a 5-year survival of 5% [12]. A study by Gutman et al. showed a median survival of 11 months in patients who underwent surgical resection [13]. In addition, surgical resection has been shown to be very effective at symptomatic palliation. A study by Hao et al. demonstrated that surgical resection for metastases to the abdomen was 100 percent palliative for their symptomatic patients [6]. For these reasons, it is important to discuss the risks and benefits of surgery with patients who qualify for it. Unfortunately, our patient's functional status was poor and he had extensive metastasis, so he was not considered a candidate for surgery. He was immediately started on immunotherapy with nivolumab prior to being enrolled into hospice care.

## 4. Conclusion

In a patient with a history of melanoma who presents with new onset or worsening anemia, metastatic melanoma should be ruled out with an EGD, colonoscopy, and capsule endoscopy if needed, regardless of how long ago the initial diagnosis or treatment of melanoma was made. Unfortunately, symptoms of gastrointestinal metastatic melanoma are nonspecific; therefore, clinical judgement should be used to decide to investigate further. Our patient was first diagnosed with melanoma 15 years prior to the time when metastasis was discovered. Early diagnosis is crucial for favorable evaluation of patients as possible surgical candidates.
